# Osteo Porosis

**Published:** 1891-06

**Authors:** A. Jasme

**Affiliations:** Savannah, Ga.


### COMMUNICATIONS.

### OSTEO POROSIS.

*Editor of The Journal of Comparative Medicine, Etc.:*

SIR,

I notice with pleasure, in THE JOURNAL OF COMPARATIVE MEDICINE AND VETERINARY ARCHIVES, a very able article on Osteo Porosis, by Dr. T. H. Berns, V. S. For some time past, it had been my intention to use the medium of your valuable Journal to, if I may so call it, open a discussion, or rather, to ask the coroboroation of my professional brothers, in a trial to throw some light upon that important subject. According to Dr. Berns's history of the disease, osteo-porosis *seems to be a disease of comparatively new origin;* this, in my mind, together with some facts I gathered, would but confirm my opinion that the disease is of infectious origin; and, if I may be allowed another supposition, that this infection may be due to, or at least is much favored by, malarial influence. Indeed, Dr. Berns and my experience is that the disease is mostly found (if not entirely) *under certain soils and certain conditions.* In my practice in the South, I have met with a great many cases, (it is very common here) and may safely state, that in nine out of ten cases, the patient was kept in badly located, and dirt floor stables.

I do not think I can now throw any light upon the subject, all we can arrive at, is: Conclusions from our own practical knowledge; I do not doubt that, if the members of the veterinary profession would give their experience of the disease through your valuable Journal, we may arrive at some conclusions, that would furnish us with a *starting* point, founded on facts, from which at the same time, much valuable informations could be obtained.

There is one thing however in Dr. Berns' letter, that I cannot pass upon without comment, that is: *It would be folly, to attempt treatment of the disease.* From his own conclusions, I think the first step in the treatment of osteo-porosis, is removal to a healthy stable, a good pasture if possible. Then, the influence having ceased, why would we not try an alterative and tonic treatment, assisted by the best kind of food, and early stimulation to affected parts? We would, by so doing, help the system, in ridding itself of *that matter, whatever it may be, which finds its way into the body of animals.*

I do not wish to be understood as criticising Dr. Berns' valuable paper, but I am simply advocating a mode of treatment which I am now experimenting upon and which has given good success in other hands than mine, as the following explanation will show: one case was under my care for some time (about six month's ago), and after a few weeks, the unmistakable symptoms of osteo-porosis *being more developed*, I told the owner that I could do no more for the patient; (enlarged maxillaries, lame in *every* leg, and not worth $5.00 at auction). The mare I have reference to happened to be a *pet mare*, and as the owner could not decide to destroy her, I advised him to see an acquaintance of mine, who, some time before, had told me of many cures he had made *of big head*. It was done, and the mare was treated by him. For the past three month's, the mare has resumed her work (saddle), and seems as good as ever; the *the swelling of the head* has not disappeared, and is still there to show the visit of the dreadful disease. I have one case on hand, that seems to be doing well, but I am not prepared to report, as I would not take such a responsibility, without proving that the treatment has been successful in my own hands. The gentleman I have reference to, is well-known here, and has the reputation of having cured many cases of big head.

Very respectfully, yours,

Savannah, Ga. A. JASME, V. S.

